# Resident Memory B Cells in Barrier Tissues

**DOI:** 10.3389/fimmu.2022.953088

**Published:** 2022-07-18

**Authors:** Choong Man Lee, Ji Eun Oh

**Affiliations:** ^1^ Graduate School of Medical Science and Engineering, Korea Advanced Institute of Science and Technology (KAIST), Daejeon, South Korea; ^2^ BioMedical Research Center, Korea Advanced Institute of Science and Technology (KAIST), Daejeon, South Korea

**Keywords:** resident memory B cells, respiratory infection, vaccine, humoral immunity, barrier tissues, mucosal immunity

## Abstract

Epithelial barriers, which include the gastrointestinal, respiratory, and genitourinary mucosa, compose the body’s front line of defense. Since barrier tissues are persistently exposed to microbial challenges, a rapid response that can deal with diverse invading pathogens is crucial. Because B cells have been perceived as indirectly contributing to immune responses through antibody production, B cells functioning in the peripheral organs have been outside the scope of researchers. However, recent evidence supports the existence of tissue-resident memory B cells (BRMs) in the lungs. This population’s defensive response was stronger and faster than that of their circulating counterparts and could resist heterogeneous strains. With such traits, BRMs could be a promising target for vaccine design, but much about them remains to be revealed, including their locations, origin, specific markers, and the mechanisms of their establishment and maintenance. There is evidence for resident B cells in organs other than the lungs, suggesting that B cells are directly involved in the immune reactions of multiple non-lymphoid organs. This review summarizes the history of the discovery of BRMs and discusses important unresolved questions. Unique characteristics of humoral immunity that play an important role in the peripheral organs will be described briefly. Future research on B cells residing in non-lymphoid organs will provide new insights to help solve major problems regarding human health.

## Introduction

Immune memory is an important component of our body’s immune system. It enables rapid and strong responses to a pathogen by pathogen-specific memory cells. Another important protective component is the barrier tissues of the body. Mucosal barrier tissues, including the lung, intestine, skin, and female reproductive tract (FRT), etc., block pathogens from invading our body at its front line. Secretory IgAs, broadly neutralizing antibodies and neutralizing antibodies are secreted to the mucosa and bind to invading pathogens, thereby isolating these harmful organisms in the mucosa and excluding them from infecting host cells. This effector mechanism is not only efficient but is also safe because it causes less inflammatory response at the site of infection, while T cell-mediated responses usually cause collateral damage to the host (1).

During infection, mature naïve B cells specific to the pathogen can enter one of four differentiation fates (2, 3). In the earlier stages of immune response, the extrafollicular response generates short-lived antibody-secreting cells (ASCs) and germinal center (GC)–independent memory B cells (MBCs) that have undergone class-switch recombination but have minimal somatic hypermutation. The extrafollicular response is in charge of the early response against influenza virus, but it also the major mechanism protecting against several pathogens, including malaria and *Salmonella* (4). In the GC, a repetitive course of affinity maturation produces plasmablasts (PBs) and MBCs that synthesize high-affinity immunoglobulins. PBs migrate to the bone marrow (BM) where they differentiate into long-lived plasma cells (PCs), but MBCs circulate through the body until they re-encounter the specific antigen. These MBCs have the capacity to re-enter the GCs or generate ASCs, providing a rapid and stronger immunity for defense upon secondary infection (5).

Since the major basis of B-cell immunity is circulating antibodies, it was speculated that there is no need for resident MBCs in the peripheral tissues. Moreover, for proper antibody production, support from GC reactions should be provided. As GCs is a complex system built on the collaborative interactions of special types of stromal cells and immune cells, it is reasonable to question whether B cells in the mucosal tissues have access to this support. Therefore, in contrast to the research on T cells, there are few studies considering the concept of tissue-resident B cells.

A recent study provided direct evidence for the existence of resident memory B cells (BRMs) in the lungs, but no direct evidence supporting BRMs in other organs has been reported (6). In this review, we will skim through the history of the discovery of BRMs and the humoral immunity of non-lymphoid barrier tissues. The probability of the existence of BRMs in non-lymphoid organs other than the lungs will be examined. Last, in anticipation of identifying BRM-specific markers, markers for MBCs and their tissue residency will be reviewed in comparison with those of resident memory T cells (TRM).

## Tissue-Resident Memory B Cells

### Evidence for Tissue-Resident Cells

It is now evident that some lymphocyte subsets are present *in situ* in non-lymphoid tissues and do not recirculate. Multiple experimental models have been used to demonstrate the residency of various cell types, including subsets of innate lymphoid cells, T cells, and recently B cells. Intravenous antibody labeling (iv-labeling) is a method that captures cells in circulation (7). Antibodies are injected intravenously into a mouse a few minutes before euthanasia. Circulating cells are captured by the antibodies but cells situated in the tissue are protected from them, allowing cells in each compartment to be distinguished from one another. The parabiosis model directly demonstrates the residency of the sessile cells. It is created by surgically joining two mice expressing distinct alleles. Circulating cells reach an equilibrium in both parabionts through the conjoined circulatory system, but the tissue-resident cells stay in the tissue, demonstrating that they reside in the tissue and do not recirculate (8–10). When infected tissues containing primed resident cells that express congenic markers are transplanted to naïve organisms, the primed resident cells do not recirculate to the recipient. Upon reactivation, local cells undergo secondary restimulation exclusively in the grafts, without the involvement of the recipient (11, 12). Models in which circulating lymphocytes have been ablated with cell type-specific antibodies have also been used (8, 13).

Based on studies using these experimental methods, the paradigm of TRM was established and intensively investigated over a decade. In contrast to TRMs, the history of BRMs is short and began with direct experimental evidence for resident non-circulating MBCs in the respiratory system (6). Since this discovery, subsequent studies have elucidated the unique characteristics of BRMs, and B cells in the human peripheral organs with resident phenotypes have been reported as well.

### Brief History of BRMs

Only recently was a subset of B cells acknowledged to be resident in the lungs, but the notion of MBCs at the periphery that are distinct from the circulating MBCs and that do not recirculate is not new (**Table 1**). In 2008, an analysis of the dispersion of MBCs after local influenza infection was reported (14). In the analysis, after intranasal influenza virus infection, the distribution of MBCs in the blood, lung, and lymphoid tissues including mediastinal lymph nodes (mLNs), Peyer’s patches, and spleen was examined. Among multiple organs, MBCs were found in the lymphoid tissues of the respiratory system, namely the mLNs and nasal-associated lymphoid tissue. Interestingly, a number of MBCs comparable to that in the mLNs were found in lungs 9 days post-infection. These lung MBCs persisted for at least 84 days, suggesting the establishment of peripheral MBCs in response to local antigen encounters. The authors anticipated the existence of mechanisms for tissue homing and delayed egress resembling those of TRMs. A few years later, more focused examinations of lung MBCs were performed. Cells situated in the lungs were separated from circulating cells by perfusing the right ventricle (RV) with PBS to clear the lungs of blood (15). Lungs harvested from influenza-infected mice after 160 days of infection still possessed MBC cells. When isolated MBCs were transferred into *scid* mice, lung MBCs outperformed MBCs derived from the mLN and the spleen in clearing the virus. These cells expressed higher levels of CD69, CXCR3, and IgA compared with MBCs in the mLNs and the spleen. These data suggest that lung MBCs are imprinted to migrate to the lungs and stay there. Next, the cross-reactive nature of lung MBCs was revealed, and it was shown that local lung GCs were responsible for supplying these cells (16). As antigenic drift is the main problem in confronting influenza virus infection, this result shows the importance of local lung mucosal immunity in defending against the infection. These early studies demonstrated interesting characteristics of a novel B-cell subset in the periphery but did not provide direct evidence for MBCs that are sessile in the lungs.

**Table 1:** Brief history of BRM research.

2008	2012	2015	2019	2020	2021	2022
MBCs are distributed in the lungs	Lung MBCs discovered	Cross-reactive nature of lung MBCs	Lung BRMs that do not recirculate	Gut MBCs with resident phenotype	BRMs, a common feature of infected lungs	BRMs, a transcriptionally & functionally distinct B cell subset
						
MBCs remained in the lungs beyond 12 weeks post infection.	Lung MBCs were isolated by RV perfusion.	Many cross-reactive lung MBCs originate from lung GCs, which show distinct selection features.	Non-circulating BRMs were discovered through a parabiosis model and iv-labeling.	Majority of human gut CD27^+^ MBCs were CD45RB and CD69 double positive.	BRMs form in *S. pneumoniae* infection.	Human and murine BRMs have a transcriptional profile distinct from that of MBCs in PBMC and SLOs.
						
	When transferred into *scid* mice, the subset migrated to the lungs and was superior in resisting secondary viral challenge.		BRMs are independent from their circulating counterparts.	Gene sets of lung CD4 and CD8 TRMs were enriched in gut MBCs.	BRMs are also found in the pneumococcal pneumonia patients.	BRMs are the main source of respiratory IgAs.
					
			BRMs are established upon encountering local antigen.			BRMs migrate to sites of inflammation and differentiate into PCs.
						
Joo et al., 2008	Onodera et al., 2012	Adachi et al., 2015	Allie et al., 2019	Weisel et al., 2020	Barker al., 2021	Mathew et al., 2021 Oh et al., 2021 Tan et al., 2022 Maclean et al., 2022

In 2019, through a parabiosis model and iv-labeling, lung MBCs were identified as a resident subset of cells in the lungs (6). In this study, resident lung MBCs were established upon local antigen infection, but not through systemic immunization, and did not access the circulation of the parabiont. Also, this cell population was preserved *in situ* when the provision of B cells from the circulation was blocked by fingolimod (FTY720), implying the independence of the subset from the circulation. Functionally, these resident B cells differentiated rapidly into ASCs during secondary infection, providing a rapid antibody response against the pathogen. These results suggest that BRMs are a key component in mucosal humoral immunity. Local resident MBCs have also been shown to contribute to the secretion of local IgA (17). Importantly, mice with these cells showed superior protection against both the homologous and heterologous strains of influenza virus, supporting the cross-reactivity of local humoral immunity.

Following BRMs’ identification as resident lymphocytes, diverse aspects of their biology have been investigated. First, BRMs are not a pathogen-specific cell population: establishment of BRMs in the lungs is detected in the *S. pneumococcus*–infected model as well as influenza virus infection (18). Second, MBCs in human lungs and gut with resident phenotypes have also been described (18–20). IgD^–^CD27^+^ MBCs from the lungs and draining LNs expressed higher levels of CD69, a representative marker for tissue-resident lymphocytes, compared with the spleen (20). When the phenotypes of CD27^+^ MBCs derived from multiple human organs including the spleen, blood, BM, LN, tonsil, and the gut were investigated, CD27^+^ MBCs in the gut included a higher percentage of CD45RB and CD69 double-positive cells (19). Also, an analysis of transcriptional profiles showed that lung MBCs cluster discretely from MBCs in lung-draining LNs or PBMCs, implying that lung BRMs have distinctive features other than the expression of CD69 (20). Markers and phenotypes of BRMs will be further discussed below.

Recently, the fate of BRMs upon secondary challenge was reported (21). In a live-imaging analysis, alveolar BRMs of influenza-infected mice were attracted by CXCL9 and CXCL10 induced by alveolar macrophages and migrated to inflammation foci to differentiate into PCs upon secondary challenge. The study not only demonstrates how BRMs react upon secondary challenge but also suggests that they interact with surrounding cells.

## Questions about Resident Memory B Cells

### Location of Resident Memory B Cells

**Figure 1:**
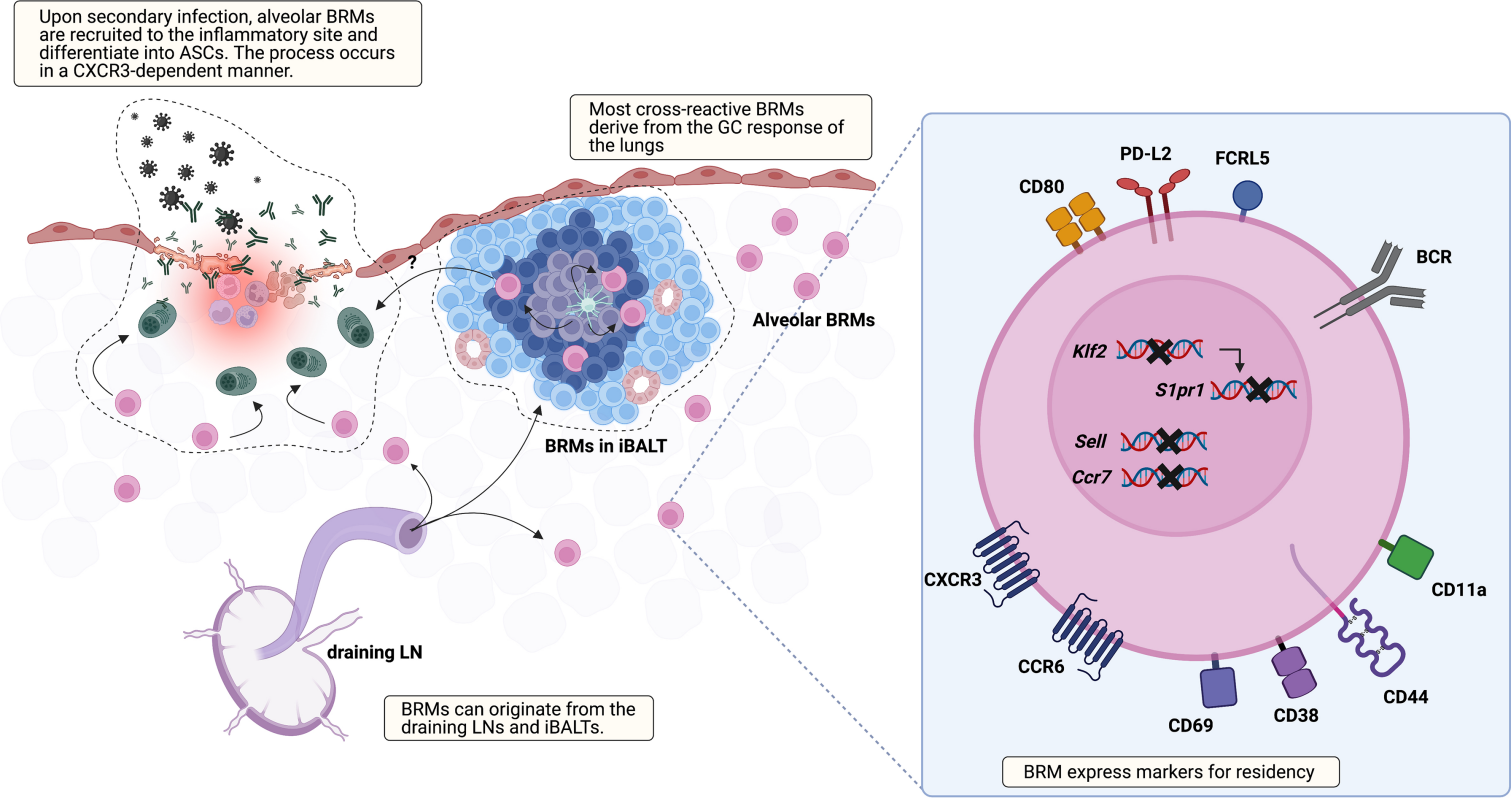
Overview of lung BRM. BRMs are established in sites exposed to local antigens. It is likely that early lung BRMs derive from the draining LNs and late cross-reactive BRMs originate from lung GC reactions, which occur in iBALTs. Lung BRMs can be located within the iBALTs or reside throughout the lung parenchyma in close contact with alveoli, independent of iBALTs. Upon secondary infection, alveolar BRMs migrate to the inflammation foci, which is mediated by alveolar macrophages, and differentiate into PCs. The corresponding functional role of BRMs in iBALT has not been reported. The illustration of the BRM shows most of the surface molecules described in published studies so far. The upregulation of CD69, which reduces surface expression of S1PR1, is consistently reported. *Klf2* is a transcription factor that mediates S1PR1 expression. The expression of genes encoding the LN homing molecules *Sell* and *Ccr7* is downregulated. CXCR3 and CCR6 facilitate recruitment and/or retention of BRM in the lung. In addition, CCR6 is related to BRM differentiation into PCs in recall response. The upregulation of CD44 and CD11a in BRM is also reported. These may serve as an adhesion molecule for BRMs, but their functional role needs to be validated. Compared with circulating MBCs, lung BRMs have been shown to express higher levels of FCRL5, CD80, and PD-L2.

Besides their presence, many aspects of BRMs are not discovered yet, including their location, markers, origin, the underlying mechanism that triggers their generation, and the environment that supports their maintenance (**Figure 1**). Regarding location, two studies using different infection models, one influenza virus and the other *S. pneumoniae*, have reported seemingly contradictory results (18, 20). The study using the influenza virus model showed that BRMs reside in the inducible bronchus-associated lymphoid tissues (iBALTs) by demonstrating the presence of antigen-specific B cells in the iBALTs beyond 110 days after infection. But BRMs were also present in an *S. pneumococcus*-infected model, in which iBALTs do not form. These are possibly complementary results, showing that BRMs not only reside in the tertiary lymphoid organs where survival niches are provided but also can persist in the bare lung parenchyma with minimum support.

A recent study reported that BRMs not situated in the iBALTs, namely alveolar BRMs, relocate themselves to the inflammatory foci upon secondary challenge in an influenza virus infection model (21). In this study, aggregates of previously activated B cells within iBALT that express tdTomato in *Aicda*(AID)^Cre/+^ Rosa26^tdTomato^ reporter mice expressed the GC B-cell marker GL7 as well. The authors also showed that the cells within iBALT-like structures display typical extensive but confined motility behavior associated with GC B-cell characteristics, suggesting that the cells in the iBALT are GC B cells, not BRMs. As previous studies suggested BRMs residing within iBALT, the question of the differences of BRMs located in each locus remains. The mechanism that supports long-standing BRMs in the lung requires further investigation, especially alveolar BRMs minimally supported by adjacent cells. This could resemble that of PCs in the gut lamina propria. A subset of lamina propria PCs live for decades and their survival is supported by surrounding cells (22). Cytokine profiling of biopsy cultures has revealed the presence of IL-6 and APRIL, which are B-cell survival factors (23). Production of these cytokines by gut epithelium, eosinophils, macrophages, and DCs may provide the survival niche for PCs (24, 25). The possibility that innate immune cells and the induced stromal cells support the survival of alveolar BRMs needs to be examined.

### Origin of Resident Memory B Cells

Given that the fate of B cells can be determined by signals provided by the surrounding tissues, the environment where BRMs are generated would affect the characteristics of BRMs (3). The GCs in the mLNs or GC-like structures of the iBALTs have been suggested as the sites for BRM generation in nasal influenza virus infection (6, 16). BRM cells are proposed to be generated in a T-cell-dependent manner at an early time point after infection (6). IgM^+^ BRMs can be identified in the lungs at day 10, but isotype-switched ones require a longer time, not exceeding 30 days. Given that GC B cells emerge earlier in the mLNs than in the lungs, it is reasonable to think that early BRMs originate from the mLNs (16). Still, specific labeling of the lungs with EdU revealed that BRMs can also arise from the lymphoid structures in the lungs (16). These results led to the proposal that early BRMs originate from B cells that migrate from the draining lymph nodes and late BRMs originate from the iBALTs (16). Questions remain as to whether iBALT-originated MBCs can access the circulation and whether B cells are determined to become BRMs during the GC reaction or if any MBCs have the capacity to become resident cells when proper conditions are provided.

Cells of different origins would have different features. Lung-specific EdU labeling has also revealed that a larger proportion of BRMs originating from the lungs are cross-reactive, in comparison with BRMs derived from the draining LN (16). This suggests that lung GC reactions are distinct from those of mLNs and that this may endow BRMs with different features (26). Fate-mapping techniques that can distinguish MBCs originating from different organs would be valuable in elucidating the heterogeneous characteristics of BRMs in the lungs.

### Markers for Resident Memory B Cells

Elucidating the nature of BRMs urgently requires specific markers. Although BRM-specific markers have not yet been discovered, they can be identified by using gating schemes combining MBC with resident markers. Classically, it has been thought that MBCs are generated from the GCs in a T-cell-dependent manner by which they lose the expression of IgM and IgD and gain somatic hypermutation (27, 28). Therefore, in both humans and mice, isotype-switched B cells have been widely accepted as a surrogate population for MBCs. But this approach can be misleading since studies have revealed that a large compartment of MBCs is generated through a route independent of GC reaction. It is evident that IgM-expressing MBCs exist (29–34). Also, though in rare cases, the presence of IgD-only and IgD/IgM double-positive MBCs has been suggested in humans (35, 36).

In humans, CD27 is expressed by most MBCs and is commonly accepted as a defining marker of this population, but this is not true in mice (35, 37). In the absence of a definitive surface marker that encompasses murine MBCs, B cells that express CD38, a surface molecule downregulated in the PC and GC B cells and have an isotype-switched phenotype are considered to be MBCs (38–40).

Systematic analysis of MBC gene expression has suggested CD80, PD-L2, and CD73 as MBC markers, and the combination of these markers divides MBCs into three major subsets (41–43). These subsets are CD80 PD-L2 double negative, PD-L2 single positive, and CD80 PD-L2 double positive. These three subsets differ in their general properties of B cells, such as isotype switching and somatic hypermutation. Regarding antibody isotypes, 95% of the CD80^-^PD-L2^-^ subset express IgM, about 40% of CD80^+^PD-L2^+^ cells have IgM, and 90% of CD80^-^PD-L2^+^ cells express IgM (43). When the BCR mutation burden was evaluated, CD80^-^PD-L2^-^ cells were less mutated, whereas 80% of CD80^+^PD-L2^+^cells had a mutated Vλ1 gene segment, and CD80^-^PD-L2^+^ cells were in between (43, 44). In line with these findings, CD80^-^PD-L2^-^ cells were found to be produced earlier in the GC reaction, around day 5 post-infection, while the production of CD80^+^ PD-L2^+^ cells dominated after 12 days, and this subset required a stronger signal from CD40-CD40L interaction with T cells. CD80^-^PD-L2^+^ MBCs peaked between these time points (34). Functional studies have revealed that three MBC subsets enter distinct routes of differentiation upon reactivation. CD80^-^PD-L2^-^ cells predominantly reenter the GC reaction and generate most of the ASCs that appear later. CD80^+^PD-L2^+^ MBCs generate IgG ASCs at an earlier time point. Again, CD80^-^PD-L2^+^ subsets are intermediate in that they can choose either route (5). Analysis of RNA expression patterns also supports this feature (5). Microarray data suggest that CD80^-^PD-L2^-^ MBCs display higher expression levels of genes encoding cell cycle–promoting molecules, and CD80^+^PD-L2^+^ MBCs express higher levels of *Zbtb32*, which is related to PC differentiation (45).

MBCs of peripheral organs express these markers as well. MBCs from the Peyer’s patches were isolated by gating CD138^-^CD9^-^CD80^+^CD73^+^ B cells (46). These markers have also been detected in BRMs in the lungs. Compared with MBCs in the mLN and spleen, lung BRMs were found to express fewer CD73 but more PD-L2 (6). These markers are evidence of the heterogeneous nature of MBCs.

Currently, iv-labeling is used to identify resident subsets of MBCs. To find specific markers for BRMs, such as CD69 and CD103 for CD8^+^ TRMs, transcriptional profiles of both murine and human BRMs have been analyzed (17, 20, 47). From their first appearance, lung MBCs showed higher expression of CXCR3 and CD69 compared with their counterparts in the mLN and spleen. Higher expression of these two molecules has been consistently reported in subsequent studies on BRMs. This expression pattern suggests the tendency of BRMs to head toward peripheral tissues and the operation of a mechanism delaying their egress, which is also observed in TRMs. Analysis of TRM transcriptional profiles has revealed the downregulation of S1PR1, the key receptor that recognizes the egression element S1P (48–50). In the TRM the transcription factor KLF2, which mediates the expression of S1PR1, is downregulated (49), and CD69, which internalizes and degrades S1PR1, is expressed (51, 52). Similarly, in the mouse model, lung BRMs, which were iv-labeling negative, were clustered discretely from iv-labeling-positive lung MBCs and MBCs from the blood, spleen, and mLN (20). The marked expression pattern of lung BRMs was the downregulation of *Ccr7, Sell, S1pr1*, and *Klf2*, and upregulation of *Cxcr3, Ccr6, Ccr1*, and *Cd69*. In addition, BRMs in a pneumococcal pneumonia model upregulated CD11a and CD44 but downregulated CD62L, a phenotype similar to that of lung CD4 TRM cells (53, 54). A similar pattern is also observed in human organs. Upregulation of CD69 and the two chemokine receptors CXCR3 and CCR6 has been detected in CD27^+^ B cells from human lungs (18, 20). At the transcript level, downregulation of *S1PR1*, *SELL*, and *CCR7* was observed. Also, as mentioned above, MBCs in the gut are mostly CD69 positive (19). These results imply that BRMs share underlying mechanisms that are in common among lymphocytes resident in non-lymphoid organs.

Other surface markers or transcriptional regulators specific to BRMs need to be identified. CD103, a marker for CD8^+^ TRMs, is not expressed in lung BRMs (6). In the case of TRMs, several transcription factors (TFs) that regulate the development and maintenance of resident cells are known (10). Blimp-1, Hobit (a homolog of Blimp-1), and AhR promote the generation and maintenance of resident cells, while the expression of Klf2 and the T-box TFs Eomes and T-bet oppose it. Some of these TFs have an effect on B cells but in a cell-type-specific manner (3, 55, 56), and studies testing these TFs on BRM formation have yet to be reported. The fact that the transcriptional program that decides the differentiation fate of MBCs is still not fully discovered is an obstacle to identifying regulatory factors in BRM formation. However, since rapid responsiveness and cross-reactivity make BRMs a promising cell type that can aid resistance to fatal infection, the underlying transcriptional program should be thoroughly revealed in order to utilize this cell population.

## Humoral Immune Cells Situated In Non-Lymphoid Barrier Organs

### Resident Memory B Cells and Antibody-Secreting Cells in the Intestine

The intestine is a unique organ in the sense that it harbors numerous lymphoid organs, the gut-associated lymphoid tissues (GALT), from birth and is the site where active interaction with the environment shapes the humoral immunity of the region. The humoral immunity of the intestine is well described in other reviews (57, 58). After pointing out several aspects of the gut humoral immune system, this review will focus on the resident memory B cells in the gut.

Factors that promote the dominance of IgA in the mucosa-associated lymphoid tissues (MALTs) are fairly well investigated in the gut. In T-cell-dependent class-switch recombination, CD40 signaling and TGF-β play an important role. It appears that NO produced by inducible nitric-oxide synthase-expressing DCs induces the expression of TGFβRII (59). Also, DCs are major players in the T cell-independent response. These cells provide proliferation-inducing ligand (APRIL) and B cell-activating factor (BAFF) that promote IgA-specific class switching. These cells are activated by commensal microbiota through toll-like receptor signaling. Dietary factors also have an effect on the production of IgA. Retinoic acid signaling is suggested to be important in generating IgA, and short-chain fatty acids produced by gut microbiota support antibody production by controlling the metabolism of B cells (60, 61). Collectively, these results show the tight relationship between the microbiota and the humoral immunity of the gut.

**Figure 2:**
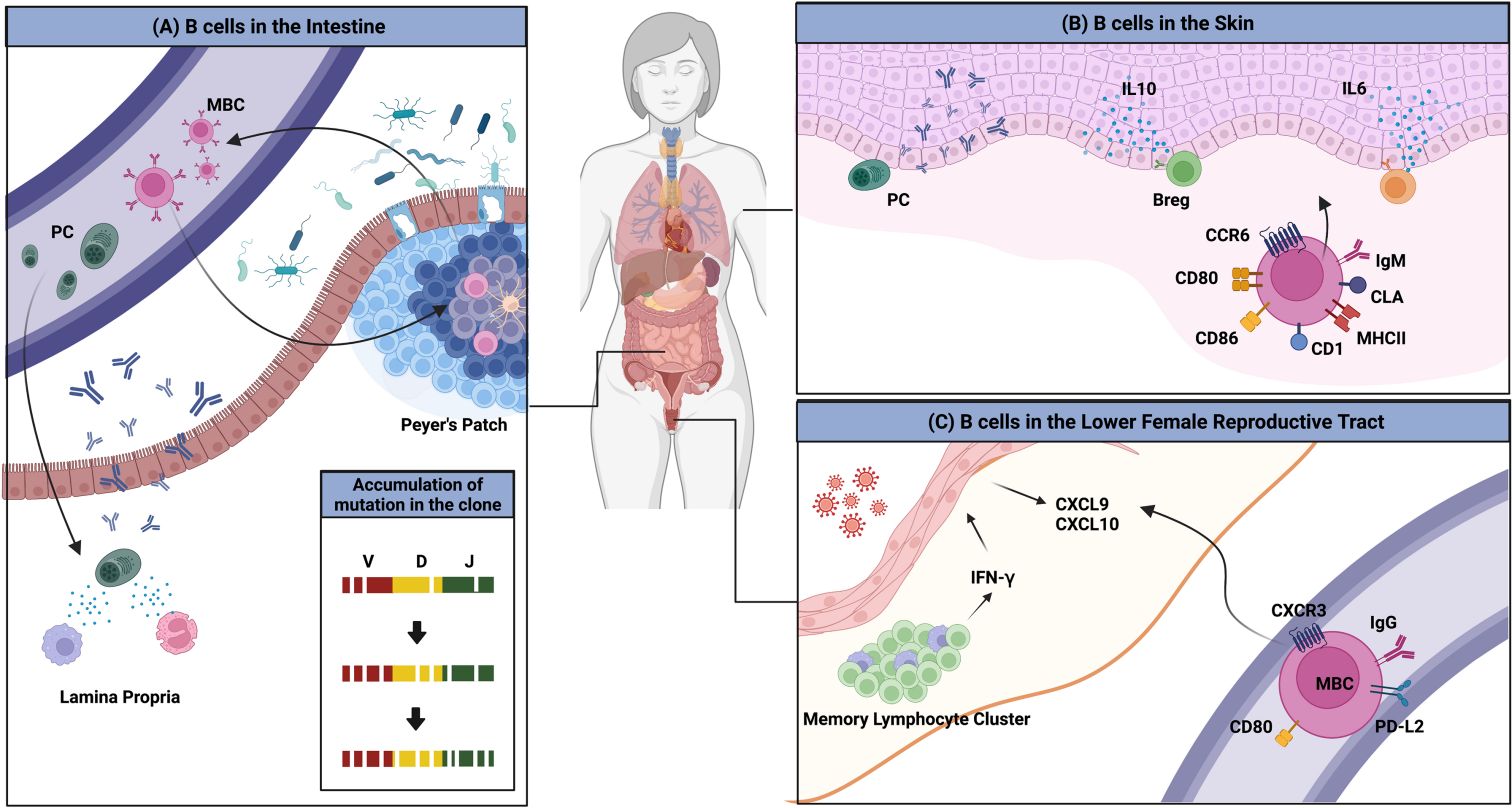
Overview of B cells in multiple peripheral organs. **(A)** The gut microbiota is a consistent stimulus to immune cells in the gut. MBCs and PCs that are formed access the circulation and return to the gut; this migration is mediated by the chemokine receptors CCR9 and CCR10, and integrin α4β7. PCs reside in the lamina propria and MBCs enter the GC reaction, which results in the evolution of BCR repertoires. **(B)** Several clues for the existence of skin-associated B cells are provided. B cells that migrate to the skin have higher expression of MHCII, CD1, CD86, CD80, and IgM. The migration to the organ is mediated by CCR6 and CLA. B cells in the skin produce antibodies locally and regulate the immune reaction directly by producing the pro-inflammatory cytokines such as IL-6 or the anti-inflammatory cytokine, IL-10. **(C)** BRMs are not established in the lower FRT. Upon secondary infection, circulating MBCs rapidly migrate to the tissues in a CXCR3-dependent manner. These cells show higher expression of MBC markers including PD-L2 and CD80. These cells could not stay long in the lower FRT tissues.

Another interesting example of the interaction between the environment and the immune system is the imprinting of GALT-derived ASCs by gut-homing molecules (**Figure 2A**). Retinoic acid secreted by DCs in the GALTs induces the expression of these molecules, which are integrin α4β7 that binds to mucosal addressin cell adhesion molecule 1 (MAdCAM-1) expressed on endothelial cells in the lamina propria, and the chemokine receptors CCR9 and CCR10, which respond to CCL25 and CCL28 produced by the intestinal epithelium (62–64). ASCs expressing these molecules home back to the gut lamina propria, where they secrete antibodies. Human IgM^hi^ transitional B cells expressing α4β7 tend to migrate to the intestine to enrich the GALTs (65). MBCs are not an exception. These molecules have been suggested to be essential for the recruitment of IgA^+^ MBCs to the intestine (66, 67).

Returned MBCs are likely to recirculate between different Peyer’s patches and re-enter the GC response, resulting in the persistence and evolution of the IgA repertoire (46). BCR repertoire analysis has revealed that PCs are more clonally related to MBCs, suggesting that MBCs recirculate. New clones were introduced upon new infection. The authors discussed this observation and proposed that this mechanism is necessary for stable interaction between the host and microbiota (46). In humans, a clonal relationship between IgM^+^ MBCs and IgA^+^ MBCs and PCs has been observed, suggesting that gut IgM^+^ MBCs can switch to expressing IgA (68). Recently, a study reported that the majority of CD19^+^CD27^+^ B cells in the human gut are CD45RB CD69 double-positive, a distinguishing feature of gut MBCs (19). CD4^+^ and CD8^+^ lung TRM gene sets were found to be enriched in this double-positive subset. These data imply the possibility that some unidentified portion of recruited MBCs may reside in the gut for a long period and operate in a unique manner.

### Skin-Associated B Cells and Their Function

The skin is the largest barrier tissue that faces a variety of daily threats, including UV, injuries, pathogens, and commensals. Traditionally, the skin was thought to lack B cells and contain mostly T cells (69, 70). However, recent studies have reported the presence of B cells with interesting functions, including antibody production and antibody-independent function, in both healthy and diseased skin (**Figure 2B**) (71).

Clonally restricted B cells have been observed in normal skin, implying the existence of B cells specific for local skin antigens (72). In normal skin of an ovine model, skin-associated B cells were found to be a heterogeneous population that comprised a B-1 B-cell–like phenotype, IgM^hi^ and CD11b^hi^, and an activated phenotype, expressing higher levels of MHC II and CD80/86 (73). IgM ASCs that reside in healthy mouse and human skin have also been observed (74). The survival of ASCs was dependent on APRIL and BAFF produced at the site. It was suggested that these B cells migrate to the skin through a CCR6-CCL20 axis (73). Cutaneous lymphocyte antigen is the molecule that guides T cells into the skin. As parenteral immunization induced its expression in ASCs, it appears that B cells are recruited to the skin in a similar manner (75). Though direct evidence of skin BRMs was not provided, these results imply their possible existence. In addition, skin-associated B cells appear to be directly involved in immune reactions in the skin.

The functions of B cells in the skin in pathologic conditions are relatively well studied. One is local antibody production. For example, pemphigus is characterized by circulating anti-desmoglein 1/3 (Dsg1/3) autoantibodies that target the desmosomal adhesion molecules anchoring epidermal keratinocytes (76). It has been suggested that Dsg1/3-specific B cells infiltrate the lesion and that autoantibodies can be produced locally (77). B cells can secrete cytokines to promote inflammation. A study using a bleomycin-induced scleroderma model reported an accumulation of IL-6–producing B cells in the inflamed skin, and the skin and lung fibrosis were attenuated in IL-6 deficient mice (78). The result demonstrates the antibody-independent function of B cells in the skin.

Regulatory B cells (Bregs) are capable of suppressing the inflammatory response by producing the anti-inflammatory cytokine IL-10. A subset of both mice and human skin–associated B cells with innate-like phenotypes, which are CD1d^hi^ CD5^+^ in mice and CD11b^+^ in humans, is reported to produce IL-10 upon stimulation (79). Bregs have been found to limit inflammation in several disease models. IL-10–deficient mice show more severe fibrosis in the scleroderma model mentioned above (78). Peritoneal B-1a cells display a regulatory function in a contact hypersensitivity model, and IL-10–producing CD1d^hi^CD5^+^ B cells can negatively regulate inflammation in an imiquimod-induced psoriasis model (80, 81).

Given the diverse role of B-cell inflammatory skin disorders, depleting pro-inflammatory B cell subsets while retaining the regulatory subset would be a promising means for treating these diseases, but the identity of Bregs is not fully elucidated. The question of whether these cells are a specific lineage or if any B cell subsets can become Bregs under certain conditions should be answered first. If the latter is the case, the conditions should be specified (82).

### Memory B Cells in the Lower Female Reproductive Tract

In terms of BRM, the lower FRT is the lungs’ opposite. Circulating antibodies are unable to enter the tissue or reach the lower FRT lumen (83, 84). Local immunization, however, can increase the titer of antibodies in the vaginal lumen, with the activity of these antibodies being higher than that of their circulating counterparts (85, 86). Also, antibodies that are passively transferred to the lumen are capable of controlling infection (87). These findings imply that antibodies in the lower FRT lumen are produced locally. This hypothesis is supported by the presence of PCs in the lower FRT of mice locally immunized with attenuated HSV type 2. These antibody-producing cells appeared under the epithelium after secondary challenge with wild-type viruses and lasted for 10 months. The increment of IgG-producing cells was more than 10 times higher than the increment of IgA-producing plasma cells, which explains why IgG is the dominant antibody isotype in the lower FRT (86). Similar results were found in a study of HIV-1: the level of vaginal secretion of anti–HIV-1 antibodies was higher than that in the serum (85). A study on SIVmac239Δnef vaccination also showed that PCs appeared after vaccination, supporting the presence of local antibody production (88).

Notably, tissues that compose the lower FRT do not permit access by circulating B cells. Immunization with attenuated HSV-2 is insufficient to establish PCs and MBCs in the tissue. In the mouse genital herpes model, only after a secondary challenge with wild-type virus were IgG^+^ circulating MBCs recruited, and they contributed to the proper antibody production (**Figure 2C**) (87). These cells express high levels of CD80, PD-L2, and CXCR3. Their migration is mediated by CXCR3-ligand chemokines induced by IFN-γ produced from CD4 TRM maintained in memory lymphoid clusters, which are immune clusters composed mainly of CD4 TRMs and macrophages (89). However, in contrast to the lung, in which BRMs are embedded for at least 120 days, BRMs do not form in the lower FRT (87). This discrepancy may result from the different microenvironments the two organs provide to B cells.

## Concluding Remarks

In this review, we have briefly described the discovery of BRMs in the lung. The timeline is short but several studies highlighting its distinguishing features have been published recently. The rapid response of BRMs upon secondary infection and their cross-reactive potential make them a valuable target for vaccine design. To control this cell population, several questions including their location, origin, specific markers, and transcriptional regulators must be answered. The different features of BRMs and their survival niches in different locations should be identified. Identifying the origin of BRMs and the cross-talk between BRMs and the microenvironment will help to determine the factors that regulate the generation and establishment of BRMs. Although there is no direct evidence of BRM existence in other barrier tissues rather than the lung, B cells and ASCs have diverse properties and play important roles in multiple barrier tissues. Further investigation is required to elucidate the characteristics and the residency features of these cells. Understanding the molecular pathways that regulate the interaction of these cells and their microenvironment could reveal the key factors that determine tissue-specific immune properties.

## Author Contributions

CL and JO wrote the manuscript. All authors contributed to the article and approved the submitted version.

## Funding

This work was supported by the National Research Foundation of Korea (NRF-2021R1C1C1004546, NRF-2021M3A9H3015689), the POSCO Science Fellowship of POSCO TJ Park Foundation (to JO) and a grant of the MD-Phd/Medical Scientist Training Program through the Korea Health Industry Development Institute (KHIDI), funded by the Ministry of Health & Welfare, Republic of Korea (to CL).

## Conflict of Interest

The authors declare that the research was conducted in the absence of any commercial or financial relationships that could be construed as a potential conflict of interest.

## Publisher’s Note

All claims expressed in this article are solely those of the authors and do not necessarily represent those of their affiliated organizations, or those of the publisher, the editors and the reviewers. Any product that may be evaluated in this article, or claim that may be made by its manufacturer, is not guaranteed or endorsed by the publisher.
